# Colour Vision Deficiency in Health Professions Education: A Narrative Literature Review

**DOI:** 10.1111/tct.70402

**Published:** 2026-03-22

**Authors:** Marnie Imhoff, Linsey Donner, Ashley Eichleberg, Kevin McGuire

**Affiliations:** ^1^ Medical Laboratory Science Program, College of Allied Health University of Nebraska Medical Center Omaha Nebraska USA

**Keywords:** colour blindness, colour vision deficiency, health professions education, healthcare professionals

## Abstract

**Background:**

Colour vision deficiency (CVD) or colour blindness can affect healthcare professionals in tasks that require colour perception. Despite its occurrence, there is limited awareness within healthcare education to support students and practitioners with CVD. This narrative literature review examines the impact of CVD on educational experiences and clinical performance, summarises recommended teaching interventions and identifies gaps in training and institutional support.

**Methods:**

A methodical search was conducted using seven databases (CINAHL, EMBASE, ERIC, Google Scholar, MEDLINE, PsycINFO and PubMed) using keywords related to CVD and healthcare education. Articles published between August 2013 and January 2026 were included if they addressed CVD in healthcare professionals or students and discussed educational practices or clinical implications. Thematic analysis was used to categorise findings and develop insights.

**Findings:**

The review identified gaps in screening, awareness and support for individuals with CVD in healthcare education and clinical practice. Few programs screen for CVD or offer accommodations for those with CVD. The literature supports the use of universal design principles, alternative instructional strategies and assistive technology. Effective interventions include grayscale imaging, high‐contrast visuals, adaptive technologies and purposeful instructional design.

**Conclusions:**

There is a need to implement changes in both healthcare education and clinical practice to support individuals with CVD. Routine screening, universal design principles, adaptive tools and standardised guidelines are essential. Future research needs to evaluate the effectiveness of current interventions, identify best practices in education and inform evidence‐based policies to promote a supportive educational environment.

## 1 Introduction

Colour vision deficiency (CVD), commonly known as colour blindness, alters an individual's ability to see colours or distinguish between different shades. The National Eye Institute indicates that those with CVD may have trouble differentiating specific shades or, in rare cases, may not perceive colour at all [1]. Colour vision deficiency, which can be either congenital or acquired, affects millions worldwide with varying prevalence by gender and ethnicity.

Healthcare professionals with CVD may experience challenges during their professional practice, particularly in situations where accurate colour detection is crucial. Many healthcare professionals may not even know they have CVD, which can make it difficult to interpret physical signs during a patient exam or when reviewing medical test results [2]. The prevalence of CVD in healthcare professionals is similar to that of the general population [3]. The incidence of CVD in females is between 0.2% and 1.7%, depending on race. The incidence is higher in males, ranging from 1.4% to 8%, also varying by race [3, 4, 5, 6, 7, 8, 9, 10].

Healthcare professionals face multiple challenges because of CVD, indicating a need for greater awareness, increased screening and better support systems to help those with CVD succeed. Health professions education needs to address these challenges to effectively prepare future professionals while maintaining high‐quality patient care. This narrative literature review summarises the existing literature and information on CVD in health profession education and its impact on clinical practice.

## 2 Methods

The review process followed a structured approach, including database selection, search strategies, inclusion and exclusion criteria, and data synthesis. To identify relevant literature, CINAHL, EMBASE, ERIC, Google Scholar, MEDLINE, PsycINFO and PubMed were searched. Keywords and search terms, listed in Table 1, were developed based on the research questions: (1) What are the best practices in teaching healthcare students with CVD? (2) How are healthcare education programs teaching students with CVD? (3) How does CVD impact healthcare professionals?

**TABLE 1:** Keywords and search terms.

Keywords and search terms
Colour blindness
Colour vision deficiency
Colour vision defect
Colour deficiency/colour blindness in laboratory science
Colour deficiency/colour blindness in allied health students
Colour deficiency/colour blindness health science students
Colour deficiency/colour blindness medical students
Colour deficiency/colour blindness health science education
Colour deficiency/colour blindness in health professionals
Teaching colour blind students
Universal design
Colour vision deficient students
Teaching technical skills to colour deficient students
Teaching microscopy to colour deficient students
Teaching microbiology to colour deficient students
Teaching chemistry to colour deficient students
Teaching haematology to colour deficient students
Interpreting urinalysis dipsticks for colour deficient students
Interpreting Gram Stains for colour deficient students
Interpreting CBC differentials for colour deficient students
Guidance for colour blind/deficient students

An initial search was conducted in August 2023, limited to English‐language publications between August 2013 and the present. The review was limited to studies published in 2013 and later to reflect current practices in education and healthcare delivery. When recent studies cited earlier sources, those original sources were also reviewed and cited as appropriate. An updated search was conducted in January 2026 to ensure coverage of recent articles on CVD background. Articles were included in the analysis if they were published in English, focused on best practices in CVD education or CVD in healthcare professionals, and provided findings, insights, or systematic reviews related to the research questions. The literature search focused broadly on healthcare professions; however, many studies did not specify participants' clinical specialties. Exclusion criteria included articles not in English, unavailable in full text or not related to the review's focus.

The relevant studies were reviewed using a thematic analysis approach to identify patterns in the literature. The process took place over several rounds, with themes becoming more defined as patterns emerged. Each article was read in full, and when similar ideas appeared across studies, they were grouped into broader themes. In keeping with recommended approaches for narrative literature reviews, the goal was not to quantify findings but to interpret and bring them together to understand what is currently known and where gaps exist [11, 12, 13, 14, 15].

## 3 Findings/Thematic Analysis

The literature addressing CVD in health professions education is organised around interrelated themes that progress from foundational understanding to educational strategies and institutional response. This review begins by describing CVD incorporating the different types of CVD and variability in colour perception. It then examines the potential impact of CVD in health professions education and clinical practice, including implications in technical skills performance, diagnostic interpretation and patient safety. To facilitate learning for those with CVD, this literature review describes classroom‐based learning interventions and technical skill‐focused strategies. However, barriers and gaps in educational support including limited awareness, absence of guidance or resources for teaching those with CVD, and inconsistent accommodations remain persistent. This findings section concludes by examining implications for educators and institutions, emphasising the need for standardised screening practices, utilisation of universal design for learning (UDL) in conjunction with other learning interventions, and policy development to support learners with CVD.

### 3.1 Understanding Colour Vision Deficiency

In humans, colour vision depends on properly functioning rods and cones in the eye. Normal colour vision depends on three types of cone photoreceptors that respond to different portions of the visible light spectrum [9]. The retina contains approximately seven million cone cells and 230 million rod cells, contributing to vision in different ways [16]. Cone cells are concentrated in the fovea, the central area of the retina, and are responsible for colour vision and vision in bright light [16]. Rods are located in the peripheral retina and do not contribute to colour vision but are responsible for vision in dim light and night conditions [16]. The cones are specialised cells that detect wavelengths of light: short wavelengths of blue (tritan), medium wavelengths of green (deutran) and long wavelengths of red (protan) [8, 9, 17]. Each cone type contains a distinct light‐sensitive protein, an opsin, encoded by a separate gene. Changes in genes encoding opsins can reduce or eliminate sensitivity to blue, red or green light, leading to CVD [16, 18, 19, 20]. When all three cones function correctly, vision is trichromatic, allowing a full range of colour perception [16, 18, 19, 20]. However, CVD occurs when one or more cones are either missing or do not function correctly [8]. According to Alamoudi [2], CVD occurs when ‘light‐sensitive cells in the retina fail to respond appropriately to variations in wavelengths of light that enable people to see an array of colors’. This condition can present as dichromacy, the complete absence of one type of cone, or anomalous trichromacy, where one type of cone is dysfunctional, leading to altered colour perception.

### 3.2 Types of Colour Vision Deficiency

Colour vision deficiency has several forms and can range from mild to severe depending on the extent of cone dysfunction. Two types of CVD that impact colour perception are dichromacy and anomalous trichromacy. Individuals with dichromacy have only two working cone cells because the third cone is either absent or not functioning correctly [3, 9, 16, 18, 19, 20]. Dichromacy has three forms: deuteranomaly, protanomaly and tritanomaly [9, 16, 18, 19, 20]. In contrast, anomalous trichromats have three cones, but one is abnormal [3, 9, 16, 18, 19, 20].

The most common form is red‐green deficiency, which affects approximately 95% of individuals with CVD [1]. There are four types of red‐green CVD: deuteranomaly, protanomaly, protanopia and deuteranopia. Deuteranomaly is the most common and occurs when the green (medium‐wavelength) cones do not function properly, making green colours appear redder [5, 19]. This type of CVD is X‐linked recessive and affects approximately 6% of males and 0.4% of females [16]. Protanomaly occurs due to the loss of red cones, and these individuals cannot detect long wavelengths of light [17]. In protanomaly, red colours are less bright and greener. Like deuteranomaly, it is X‐linked recessive, but less common, affecting approximately 1% of males and 0.1% of females [16]. Deuteranopia and protanopia are more severe forms of red‐green CVD. These cause a complete inability to distinguish between red and green. Those with protanopia see red as black, while certain shades of orange, yellow, and green may appear yellow [20]. Deuteranopia individuals may see green as beige and red as a brownish‐yellow [20].

A rare type of CVD is blue‐yellow deficiency, with only 0.01% of males and females being affected [16]. This type includes tritanomaly, where it is hard to tell blue apart from green and yellow from red. Tritanopia is more severe, where people cannot differentiate blue from green, purple from red, or yellow from pink. Both types are autosomal‐dominant conditions [19].

The rarest type of CVD is the complete inability to see colour, referred to as monochromacy or achromatopsia. People with monochromacy only see in shades of black, white and grey. In addition to being unable to see colour, these individuals may experience extreme light sensitivity and struggle to see clearly.

### 3.3 Screening for Colour Vision Deficiency: Potential Impact of Colour Vision Deficiency in Health Professionals' Education and Practice

While CVD may seem like a minor condition, its implications for health profession students and practitioners may be significant. There is a risk of clinical errors due to the inability to accurately interpret colour‐coded test results and diagnostic tools, medication labels, and patient charts, which can lead to critical errors compromising patient safety. For example, challenges in interpreting the colour of clinical laboratory‐stained slides or differentiating between shades in dermatological conditions can hinder academic performance and clinical competency. In addition, significant education barriers occur when students with CVD struggle with tasks requiring colour discrimination, leading to frustration, decreased confidence, poor academic performance, and potential attrition from health profession programs.

Screening for CVD is important in professions such as healthcare, where inaccurate colour interpretation can lead to medical errors. Several tests are available to detect CVD, ranging from the well‐known Ishihara Color Test to newer computerised methods such as the Cambridge Test [21, 22]. Choosing the appropriate screening test should be based on the purpose of screening and the known strengths and limitations of a screening test.

Spalding [3] presents a strong case for routine screening of medical students for CVD. The study findings highlight the risk of ignoring CVD in health professionals, including potential errors in clinical practice due to misinterpretation of colour‐coded information. In Spalding's [3] survey of 40 doctors, the most common difficulties experienced included recognising body colour changes (*n* = 26); identifying dermatology rashes/erythema of the skin (*n* = 25), viewing charts, slides and codes (*n* = 24); reading test‐strips for blood and urine (*n* = 18); and microscopy (*n* = 13). This study also identified a lack of awareness and institutional barriers as significant barriers for students with CVD; not one of the 40 doctors surveyed reported being given any help or advice concerning their colour deficiency by a medical teacher. The author notes screening students for CVD would enable early identification of CVD and allow implementation of appropriate accommodations such as alternative tools and teaching methods. Dhingra et al. [23] had similar recommendations for routine screening of CVD status at the time of student admission into medical school. In this study, the authors found that students with CVD made more errors interpreting colour‐dependent photographs. Students with CVD made 5–26 errors out of 75 items when examining colour‐dependent photographs (mean [SD] 13.17 [5.87]), whereas those students without CVD made 2–13 errors (mean [SD] 5.53 [3.04]) [23]. This demonstrates that students with CVD could have problems in learning histology, pathology, haematology, microbiology, dermatology, paediatrics, medicine, biochemistry, and ophthalmology. Campbell et al. [24] also support screening for CVD in medical schools because not all medical practitioners with CVD are aware of their condition and/or their severity. Screening will ensure that students with CVD are aware of their condition, allowing them to learn support strategies as students and apply them in clinical practice.

Raynor et al. [25] conducted an analysis of the guidance available to medical students and professionals with CVD. This study revealed significant gaps in the support structures available for individuals entering health professions, where tasks such as interpreting colour‐coded charts, diagnostic images, and test results are essential. For example, students with CVD may struggle to differentiate between red and green signals in arterial blood gas results, potentially leading to misinterpretation. The authors also note that doctors and medical students with CVD may be less capable and confident at identifying various scenarios, which could affect patient outcomes. Table 2 provides a summary and examples of the impact CVD may have on health professions education and practice. The current lack of guidance for both students and healthcare educators not only limits career progression within healthcare but also increases the risk of clinical errors, potentially compromising patient care.

**TABLE 2:** Impact of colour vision deficiency (CVD) on health professions education and practice [3, 25].

Impact of CVD	Examples
Difficulty distinguishing colour‐coded information	Difficulty reading medication colour labels, solution caps or test tube stoppers
Inability or difficulty interpreting diagnostic tests	Difficulty interpreting Gram stains, urinalysis dipsticks, pH indicators or other colour‐based assay results
Inability or reduced ability to recognise colour‐related clinical signs	Difficulty identifying cyanosis, jaundice, erythema, bile staining or blood staining

### 3.4 Classroom Learning Interventions

Learning interventions in the classroom are largely shaped by the UDL and Universal Design for Instructor (UDI) Guidelines, which offer guidance for designing learning environments. ‘UDL is a framework to improve and optimize teaching and learning for all people based on scientific insights into how humans learn’ [26]. This article will not attempt to address everything mentioned in the UDL Guidelines but rather will focus on key actionable components that relate to CVD in the classroom setting found in the relevant literature. For a complete list of the UDL Guidelines, visit the CAST website [26].

Several UDL Guidelines designed to support students with CVD in the classroom may also translate to work environments, including the laboratory and clinical setting. Table 3 includes a list of recommendations found throughout the literature for classroom learning interventions. While the UDL strategies presented in Table 3 may not specifically target CVD, they remain recommended best practices. These recommendations can help both CVD and non‐CVD students. For example, Landini and Perryer [33] enhanced stained histological images, and most CVD subjects in their study indicated better perception in the enhanced images compared to the original. In addition, they found that those without CVD preferred the enhanced images, which indicates that the enhancements aided those with CVD without hindering those without CVD [33]. Following UDI Guidelines from the beginning can provide all learners with appropriate resources instead of having to make individual accommodations later [27, 29].

**TABLE 3:** Examples of learning interventions.

**Classroom learning interventions**
Presentations/graphics/text 1. Use black font and lines with contrasting white background for slides, text, figures, graphs, handouts, and readings [20]. Avoid dark backgrounds with red elements or red backgrounds with dark elements [27]. 2. Use black markers on whiteboards [20]. 3. Use large, sans serif font for text which helps separate letters [20]. 4. Use large text and visual images [20]. 5. Utilise a high degree of contrast, avoid using contrasting green and red colours, or low‐saturation colours with similar lightness. Instead use high saturation/pure colours [20, 28]. 6. Use wide and clear lines; avoid small or thin elements [20, 28]. 7. Use bolding, italicising or underlining to emphasise; do not rely on hue alone [20, 28]. 8. Separate points with arrows, letters, numbers or patterns rather than colour [20]. 9. Use labels or non‐colour cues in addition to colour cues [7, 20, 28]. Label colours with text [20]. 10. Use arrows, numbers or letters in black ink instead of colour to distinguish items [29]. 11. Switch laser pointers from red to green or blue [20, 29]. 12. Provide high‐quality greyscale images or prints of slides or images [20, 29]. 13. Test presentations before presenting [28].
Assistive technology 14. Utilise assistive technology, such as coloured overlays, tools to modify colour in images (FIGI, Adobe Photoshop, colorblind.org, Chrome Daltonize, Mozilla Firefox extension, Oxy‐Iso lenses, DanKam) or glasses with high‐contrast colour lenses for students with CVD [16, 20, 29]. 15. Utilise Learning Management System platforms such as Canvas or Blackboard, which offer settings for better accessibility [20].
Physical setting 16. Move seats closer to screens and boards [20]. 17. Utilise bright lighting with no glare or shadows on boards or screens [20].
Instructor 18. Ask students with CVD what practices have helped or would help them learn [20]. 19. List in syllabi for students with CVD to consult with the instructor to discuss any assistance that could improve learning [20]. 20 Use small group projects or demonstrations for how the ‘normal’ colour palette may be perceived by persons with CVD explore online colour blindness ‘simulators’ [20].

Learning interventions related to technical skills
Technology 1. ‘Titration ColorCam’ is an Android‐based application that utilises a smartphone's camera to capture and analyse colour changes during a titration experiment, providing users with audio and tactile feedback [30]. 2. Automated titrations with text‐to‐speech capabilities utilising an Arduino microcontroller with a Bluetooth‐enabled 1Sheeld+ application [31]. 3. The Color Blind Pal application allows students to use their phone cameras to identify colours by pointing at an object, freezing the screen, adjusting the target area and receiving a text‐based colour readout of the selected item [32]. 4. The ColorAssist Lite application generates a text‐based colour readout from images captured using an Apple smartphone camera [32]. 5. Visolve is computer software that enhances on‐screen colours, making them more distinguishable and tailored to an individual's colour perception [32]. 6. Photoshop and ImageJ imaging platforms can be utilised to perform hue stretching, which adjusts the range of hues in images to better align with the perceptual range of CVD observers [33]. 7. Colour deconvolution digitally separates haematoxylin and eosin‐stained sample images into two distinct images, redistributing the colour information into hues that are more easily distinguishable [33].
Focusing techniques 8. Examine morphologic changes in grayscale images as opposed to focusing on colour to identify cells and tissues [34]. 9. Observe high‐quality grayscale versions of images alongside colour images to distinguish structures that might otherwise be obscured by surrounding cells or tissue components [17]. 10. Ensure adequate lighting when examining patients, analysing images, interpreting chemical reactions or viewing specimens under a microscope [35, 36].
Instructor 11. Emphasise alternative cues, including patient history, brightness and contrast adjustments, detailed observation, tactile examination, or seeking assistance of a colleague or instructor [23]. 12. Allow additional time for students to locate non‐colour identifiers [29, 37]. 13. Stress the importance of asking for help when struggling with colour differentiation [29].

### 3.5 Learning Interventions Related to Technical Skills

Challenges related to technical skills arise in a variety of healthcare settings due to reliance on colour perception, including interpreting special stains in histology, pathology, haematology and microbiology; detecting colour changes in skin and mucosa in dermatology, paediatrics, surgery and general medicine; conducting colorimetric tests in biochemistry; and performing fundus examinations in ophthalmology [23]. Learning interventions in healthcare education settings often emphasise the development of technical skills essential for accurate diagnoses and patient care. These methods may include direct instruction, simulated environments and observation of histological and laboratory findings. The literature highlights technology tools, focusing techniques and instructor guidance to assist those with CVD in developing technical skills.

Advances in technology have introduced tools to enhance learning and mitigate challenges faced by students with CVD. Some examples of the technologies included in literature are listed in Table 3. The technologies available are primarily used for interpreting titrations or specific colours on an image [30, 31, 32, 33]. By utilising these technologies, the CVD learner can identify colour‐change reactions through impulses or adjust the colour of images to the individual's preference [30, 31, 32, 33].

In addition to utilising technology, focusing techniques can help students in healthcare education who are CVD. These strategies are included in Table 3. Providing students with greyscale images allows them to focus on specific structures and components to identify cells and abnormalities [17, 34]. This provides students with the opportunity to compare the greyscale image to the colour image to develop their own strategies to aid in identification. Jose [38], a physician with CVD, noted that relying on structure, detail and pattern recognition, not colour, as cues is an important alternative measure for people with CVD. Another focusing technique is ensuring adequate lighting when examining patients, looking at images and operation of a microscope [35, 36]. This allows students optimal viewing conditions and the ability to examine other characteristics outside of colour. Research on environmental factors influencing colour perception in individuals with CVD shows that lighting affects how well they interpret colours. Abdurachman and Adhitama [39] found that moderate indoor lighting, ranging from 300 to 500 lx, supported the most accurate and efficient colour difference identification among adults with red‐green CVD, with performance declining under both dim and overly bright conditions. Kameyama et al. [40] also demonstrated that lighting characteristics influence visual recognition, although their study was conducted with individuals with normal colour vision. Their findings showed that the spectral quality of illumination impacts how biological structures are perceived. In their study, blue light enhanced recognition of fine structures such as exposed blood vessels and nerves, red light improved visibility of deeper structures beneath intact skin, and white light provided the best visibility when broader anatomical discrimination was necessary [40]. The findings from both these studies suggest that the visual environment may influence some challenges associated with CVD, and that both lighting colour and intensity can affect accuracy and efficiency in colour‐dependent tasks. Using appropriate and balanced lighting could be helpful in supporting learners and practitioners with CVD in educational, laboratory and clinical settings.

Instructors can help students who are CVD by incorporating the strategies listed in Table 3. Encouraging students to seek peer support or professional input further enhances diagnostic accuracy. Dhingra et al. [23] recommended emphasising history‐taking, adjusting brightness and contrast to differentiate colours, closely observing details, using touch to assess skin rashes, and telling students to seek assistance when needed. Meeks [29] also advised students with CVD to inform their instructors, enabling the development of tailored strategies. These strategies might include additional support during clinical training and CVD screening through student health services.

## 4 Discussion

### 4.1 Key Findings

Individuals with CVD face barriers in education, training and clinical practice, often due to a lack of awareness and institutional support. The impact of CVD on individuals in health professions needs to be addressed within educational and professional contexts. There is a need for accommodation, including universal design principles, alternative tools and proactive guidelines, which are essential to support individuals with CVD and ensure equitable learning. Routine screening, early identification and structured support systems are necessary to address the challenges posed by CVD in health professions education.

### 4.2 Barriers and Gaps in Education

There are several gaps in both education and clinical practice for individuals with CVD. Despite evidence of the challenges they face, comprehensive resources and training programs tailored to their needs remain limited. For example, many health profession programs do not provide simulation tools or practical exercises designed for colour‐deficient students. There is limited use of inclusive teaching tools used in health profession education. While greyscale and alternative diagnostic tools have shown promise, few institutions currently provide these alternatives for interpreting colour‐coded slides or laboratory tests.

In addition to these educational gaps, existing policies and advice for students and practitioners with CVD are inconsistent, resulting in inadequate support. Some educational programs or healthcare facilities screen for CVD but fail to offer accommodations or adaptive tools for those identified as colour deficient. The lack of support extends to those educating individuals with CVD. Most institutions do not provide guidance or resources on how to screen for CVD, nor how to teach students with colour vision deficiency.

These gaps are highlighted in experiences shared by healthcare professionals with CVD. Spalding et al. [35] discussed the challenges faced by medical students and practitioners with CVD. They note the lack of resources and tailored advice for colour‐deficient individuals. For example, one optometrist with abnormal colour vision could not see the redness of inflammation of the conjunctiva or optic nerve head. This inability to see the coloured signs of illness led to significant anxiety about the risk of error and diminished confidence. Spalding's [41] earlier work offers a personal perspective of a colourblind physician, revealing the experiences of navigating a colour‐dependent profession. He describes a situation where he misidentified the colour of a patient's rash, leading to an initial misdiagnosis. The difficulties of medical professionals with CVD relate to tasks where colour is a critical diagnostic indicator was recognised in an article by Spalding [3] (as cited in Voke [42]) in which a range of practitioners with CVD including a physician, surgeon, anaesthesiologist, endoscopist, ophthalmologist and a nurse reported difficulties in identifying organs, the presence of pus, blood, cyanosis, jaundice and facial discolouration. Jose [38] shared his perspective on the importance of physicians recognising their CVD to improve patient safety and reduce diagnostic errors. Jose shared that he cannot see cyanosis or examine the ears, eyes, or skin but has learned to ask for help from colleagues with competent colour vision to confirm clinical observations. These accounts demonstrate a critical gap: the medical field's limited acknowledgement of the challenges encountered by individuals with CVD, leading to feelings of inadequacy and self‐doubt.

Campbell et al. [24] explored the effect of abnormal colour vision on identifying clinical signs from vomit or stool and interpreting stained Ziehl‐Neelsen sputum smear slides. Their findings revealed significant deficits in diagnostic accuracy and speed in individuals with CVD. Their findings showed that participants with CVD were 50% less likely to correctly identify the red‐stained acid‐fast bacilli using a microscope than those with normal colour vision. Similarly, Poole et al. [43] investigated the interpretation of histopathology slides among individuals with CVD. The study demonstrated a discrepancy in diagnostic performance, with participants unable to differentiate between eosinophilic and basophilic staining patterns in tissue specimens. One participant misinterpreted a malignancy due to their inability to distinguish between subtle colour variations. These findings demonstrate the necessity of developing inclusive teaching methods and diagnostic aids to ensure equitable education and clinical competency.

A recent survey study of CVD in Anatomic Pathology found that practicing pathologists and cytologists with CVD developed effective approaches that did not compromise diagnostic interpretations [44]. This study shows that with the proper support and strategies, individuals with CVD can adapt and perform successfully. Educators could apply these pedagogical approaches to help CVD students find ways to overcome perceived barriers.

### 4.3 Implication for Educators and Institutions

While the list of classroom interventions in Table 3 is not all‐inclusive, it provides a starting point for best practices regarding classroom learning interventions for both students with and without CVD in health professions programs. It would be beneficial to explore current interventions healthcare programs are utilising and gather feedback from those with CVD about which practices are most effective depending on the type of CVD.

Effective learning interventions for technical skills in clinical and laboratory environments must incorporate both innovative and adaptive strategies. By addressing diverse learning needs through interventions outlined in Table 3, educators can create inclusive, supportive environments that enhance both learning outcomes and patient safety. Several studies have examined strategies to aid teaching those with CVD, offering valuable insights into designing better support for students with CVD.

Rubin et al. [17] examined the use of grayscale images in teaching histology to colour‐deficient medical students. While the grayscale images improved accessibility, the study noted a lack of widespread adoption of such methods. For example, a student with CVD reported feeling excluded during lectures where slide interpretation relied solely on colour‐coded staining without the alternative grey‐scale images. The authors advocate for integrating universally accessible teaching tools, but there has been limited progress in inclusive curriculum design at many institutions.

Raynor et al. [25] found existing advice inconsistent and often insufficient to address the challenges faced by medical students and physicians with CVD. They noted one medical school provided no accommodations for colour‐deficient students during clinical examinations, leading to difficulties interpreting colour‐coded diagnostic charts and drug labels. This study recommends standardised policies and resources to support students and practitioners in navigating their professional responsibilities.

Meeks et al. [29] also advocate for the adoption of UDL principles to support students with CVD in medical education. The authors emphasise the importance of early identification and intervention because individuals are more receptive and open to advice when actively learning.

Raynor et al.’s [25] survey sent to 33 United Kingdom medical schools with responses from 30 showed that 16.7% (*n* = 5) screen for CVD and only 10% (*n* = 3) offer formal guidance for medical students with CVD. The authors recommend clearer guidelines and proactive measures to address the challenges. They suggest the use of alternative diagnostic tools and the implementation of UDL principles when teaching students with CVD, which will enhance student learning to improve confidence and accuracy.

These studies demonstrate the need for consistent educational practices that address the needs of students and practitioners with CVD. There is also a need for ongoing evaluation of interventions and policies to ensure health professions education supports the success of future healthcare professionals.

## 5 Conclusion and Future Research

There is a need for systemic changes in health professions education and clinical practice to support individuals with CVD. By integrating inclusive teaching tools, standardising guidance and fostering a supportive environment, healthcare educators can ensure equitable opportunities for all students and future practitioners.

Future research to address the gaps identified in the literature should focus on the following: (1) determine current learning interventions for teaching individuals with CVD; (2) evaluate inclusive teaching methods and the impact of these methods on academic and clinical performance; (3) identify best practices for accommodating individuals with CVD in health professions education to allow for standardisation of accommodations; (4) develop adaptive diagnostic tools such as digital enhancements for colour‐coded data or alternative imaging techniques; and (5) create evidence‐based recommendations for policies that promote inclusivity and support students with CVD.

## Author Contributions

**Marnie Imhoff:** conceptualization, investigation, writing – original draft, writing – review and editing, visualization, methodology, formal analysis, resources. **Linsey Donner:** conceptualization, investigation, writing – original draft, writing – review and editing, visualization, formal analysis, resources. **Ashley Eichleberg:** conceptualization, investigation, writing – original draft, writing – review and editing, visualization, formal analysis, resources. **Kevin McGuire:** conceptualization, investigation, writing – original draft, writing – review and editing, visualization, formal analysis, resources.

## Funding

The authors have nothing to report.

## Ethics Statement

This narrative review does not require approval because it evaluates publicly available information.

## Conflicts of Interest

The authors declare no conflicts of interest.

## Permission to Reproduce Material From Other Source

No reproduced material was used in the article.

## Data Availability

The data that support the findings of this study are available from the corresponding author upon reasonable request.
